# Supplementary material for: Multiple Targets for Oxysterols in Their Regulation of the Immune System
Source: Cells. 2021 Aug 13;10(8):2078. doi: 10.3390/cells10082078 (PMC8391951; doi:10.3390/cells10082078)
Supplement: Supplementary file 1 [file cells-10-02078-s001.zip › Supplemental Figure 2 V2.pdf]

	GPR183	*GPR17*	*CXCR2*	*SMO*	*Ligand gated*	*Voltage gated (196)*	*Multi-Stimuli*
cholesterol				(Cooper et al. 2003; Huang et al. 2016; Xiao et al. 2017)	153		
1,25-OHC (VitD)					154		
7a-OHC	(Hannedouche et al. 2011; Liu et al. 2011)	(Sensi et al. 2014)					
7k-C					156	192;197	215.1, 221.1
19-HC			(Raccosta et al. 2013)				
20(S)-HC				(Corcoran and Scott 2006; Dwyer et al. 2007; Myers et al. 2013; Nachtergaele et al. 2012)			214
22(S)-HC				(Corcoran and Scott 2006; Dwyer et al. 2007)			
22(R)-HC		(Sensi et al. 2014)	(Raccosta et al. 2013)				214
24(S)-HC			(Raccosta et al. 2013)	(Corcoran and Scott 2006)	176-180, 182	192	214
24(R)-HC							84
24k-C							
24(S),25-EC				(Raleigh et al. 2018)			
25-HC	(Hannedouche et al. 2011; Liu et al. 2011)		(Raccosta et al. 2013)	(Corcoran and Scott 2006)	155,156; 181,182		214
27-HC		(Sensi et al. 2014)	(Baek et al. 2017; Raccosta et al. 2013)		183		214
7α,25-OHC	(Hannedouche et al. 2011; Liu et al. 2011; Lu et al. 2017)						
7β,25-OHC	(Hannedouche et al. 2011; Liu et al. 2011)						
7k,25-OHC				(Myers et al. 2013)			
7α,27-OHC	(Hannedouche et al. 2011; Liu et al. 2011; Lu et al. 2017)						
7β,27-OHC	(Liu et al. 2011)			(Raleigh et al. 2018)			
7k,27-OHC				(Myers et al. 2013)			
triol						198,(199)	

Baek, Amy E., Yen Rei A. Yu, Sisi He, Suzanne E. Wardell, Ching Yi Chang, Sanghoon Kwon, Ruchita V Pillai, Hannah B. McDowell, J. Will Thompson, Laura G. Dubois, Patrick M. Sullivan, Jongsook K. Kemper, Michael D. Gunn, Donald P. McDonnell, and Erik R. Nelson. 2017. "The Cholesterol Metabolite 27 Hydroxycholesterol Facilitates Breast

Cancer Metastasis through Its Actions on Immune Cells." *Nature Communications* 8(1):1–10. doi: 10.1038/s41467-017-00910-z.

Cooper, Michael K., Christopher A. Wassif, Patrycja A. Krakowiak, Jussi Taipale, Ruoyu Gong, Richard I. Kelley, Forbes D. Porter, and Philip A. Beachy. 2003. "A Defective Response to Hedgehog Signaling in Disorders of Cholesterol Biosynthesis." *Nature Genetics* 33(4):508–13. doi: 10.1038/ng1134.

Corcoran, Ryan B., and Matthew P. Scott. 2006. "Oxysterols Stimulate Sonic Hedgehog Signal Transduction and Proliferation of Medulloblastoma Cells." *Proceedings of the National Academy of Sciences of the United States of America* 103(22):8408–13. doi: 10.1073/pnas.0602852103.

Dwyer, Jennifer R., Navdar Sever, Marc Carlson, Stanley F. Nelson, Philip A. Beachy, and Farhad Parhami. 2007. "Oxysterols Are Novel Activators of the Hedgehog Signaling Pathway in Pluripotent Mesenchymal Cells." *Journal of Biological Chemistry* 282(12):8959–68. doi: 10.1074/jbc.M611741200.

Hannedouche, Sébastien, Juan Zhang, Tangsheng Yi, Weijun Shen, Deborah Nguyen, João P. Pereira, Danilo Guerini, Birgit U. Baumgarten, Silvio Roggo, Ben Wen, Richard Knochenmuss, Sophie Noël, Francois Gessier, Lisa M. Kelly, Mirka Vanek, Stephane Laurent, Inga Preuss, Charlotte Miault, Isabelle Christen, Ratna Karuna, Wei Li, Dong In Koo, Thomas Suply, Christian Schmedt, Eric C. Peters, Rocco Falchetto, Andreas Katopodis, Carsten Spanka, Marie Odile Roy, Michel Detheux, Yu Alice Chen, Peter G. Schultz, Charles Y. Cho, Klaus Seuwen, Jason G. Cyster, and Andreas W. Sailer. 2011. "Oxysterols Direct Immune Cell Migration via EBI2." *Nature* 475(7357):524–27. doi: 10.1038/nature10280.

Huang, Pengxiang, Daniel Nedelcu, Miyako Watanabe, Cindy Jao, Youngchang Kim, Jing Liu, and Adrian Salic. 2016. "Cellular Cholesterol Directly Activates Smoothened in Hedgehog Signaling." *Cell* 166(5):1176-1187.e14. doi: 10.1016/j.cell.2016.08.003.

Liu, Changlu, Xia V. Yang, Jiejun Wu, Chester Kuei, Neelakandha S. Mani, Li Zhang, Jingxue Yu, Steven W. Sutton, Ning Qin, Homayon Banie, Lars Karlsson, Siquan Sun, and Timothy W. Lovenberg. 2011. "Oxysterols Direct B-Cell Migration through EBI2." *Nature* 475(7357):519–23. doi: 10.1038/nature10226.

Lu, Erick, Eric V. Dang, Jeffrey G. McDonald, and Jason G. Cyster. 2017. "Distinct Oxysterol Requirements for Positioning Naïve and Activated Dendritic Cells in the Spleen." *Science Immunology* 2(10). doi: 10.1126/sciimmunol.aal5237.

Myers, Benjamin R., Navdar Sever, Yong Chun Chong, James Kim, Jitendra D. Belani, Scott Rychnovsky, J. Fernando Bazan, and Philip A. Beachy. 2013. "Hedgehog Pathway Modulation by Multiple Lipid Binding Sites on the Smoothened Effector of Signal Response." *Developmental Cell* 26(4):346–57. doi: 10.1016/j.devcel.2013.07.015.

Nachtergaele, Sigrid, Laurel K. Mydock, Kathiresan Krishnan, Jayan Rammohan, Paul H. Schlesinger, Douglas F. Covey, and Rajat Rohatgi. 2012. "Oxysterols Are Allosteric Activators of the Oncoprotein Smoothened." *Nature Chemical Biology* 8(2):211–20. doi: 10.1038/nchembio.765.

Raccosta, Laura, Raffaella Fontana, Daniela Maggioni, Claudia Lanterna, Eduardo J. Villablanca, Aida Paniccia, Andrea Musumeci, Elena Chiricozzi, Maria Letizia Trincavelli, Simona Daniele, Claudia Martini, Jan Ake Gustafsson, Claudio Doglioni, Safiyè Gonzalvo Feo, Andrea Leiva, Maria Grazia Ciampa, Laura Mauri, Cristina Sensi, Alessandro Prinetti, Ivano Eberini, J. Rodrigo Mora, Claudio Bordignon, Knut R. Steffensen, Sandro Sonnino, Silvano Sozzani, Catia Traversari, and Vincenzo Russo. 2013. "The Oxysterol-Cxcr2 Axis Plays a Key Role in the Recruitment of Tumor-Promoting Neutrophils." *Journal of Experimental Medicine* 210(9):1711–28. doi: 10.1084/jem.20130440.

Raleigh, David R., Navdar Sever, Pervinder K. Choksi, Monika Abedin Sigg, Kelly M. Hines, Bonne M. Thompson, Daniel Elnatan, Priyadarshini Jaishankar, Paola Bisignano, Francesc R. Garcia-Gonzalo, Alexis Leigh Krup, Markus Eberl, Eamon F. X. Byrne, Christian Siebold, Sunny Y. Wong, Adam R. Renslo, Michael Grabe, Jeffrey G. McDonald, Libin Xu, Philip A. Beachy, and Jeremy F. Reiter. 2018. "Cilia-Associated Oxysterols Activate Smoothened." *Molecular Cell* 72(2):316-327.e5. doi: 10.1016/j.molcel.2018.08.034.

Sensi, Cristina, Simona Daniele, Chiara Parravicini, Elisa Zappelli, Vincenzo Russo, Maria L. Trincavelli, Claudia Martini, Maria P. Abbracchio, and Ivano Eberini. 2014. "Oxysterols Act as Promiscuous Ligands of Class-A GPCRs: In Silico Molecular Modeling and in Vitro Validation." *Cellular Signalling* 26(12):2614–20. doi: 10.1016/j.cellsig.2014.08.003.

Xiao, Xu, Jing Jie Tang, Chao Peng, Yan Wang, Lin Fu, Zhi Ping Qiu, Yue Xiong, Lian Fang Yang, Hai Wei Cui, Xiao Long He,

Lei Yin, Wei Qi, Catherine C. L. Wong, Yun Zhao, Bo Liang Li, Wen Wei Qiu, and Bao Liang Song. 2017. "Cholesterol Modification of Smoothened Is Required for Hedgehog Signaling." *Molecular Cell* 66(1):154-162.e10. doi: 10.1016/j.molcel.2017.02.015.
