# Supplementary figures and images for: Multiple Targets for Oxysterols in Their Regulation of the Immune System
Source: Cells. 2021 Aug 13;10(8):2078. doi: 10.3390/cells10082078 (PMC8391951; doi:10.3390/cells10082078)

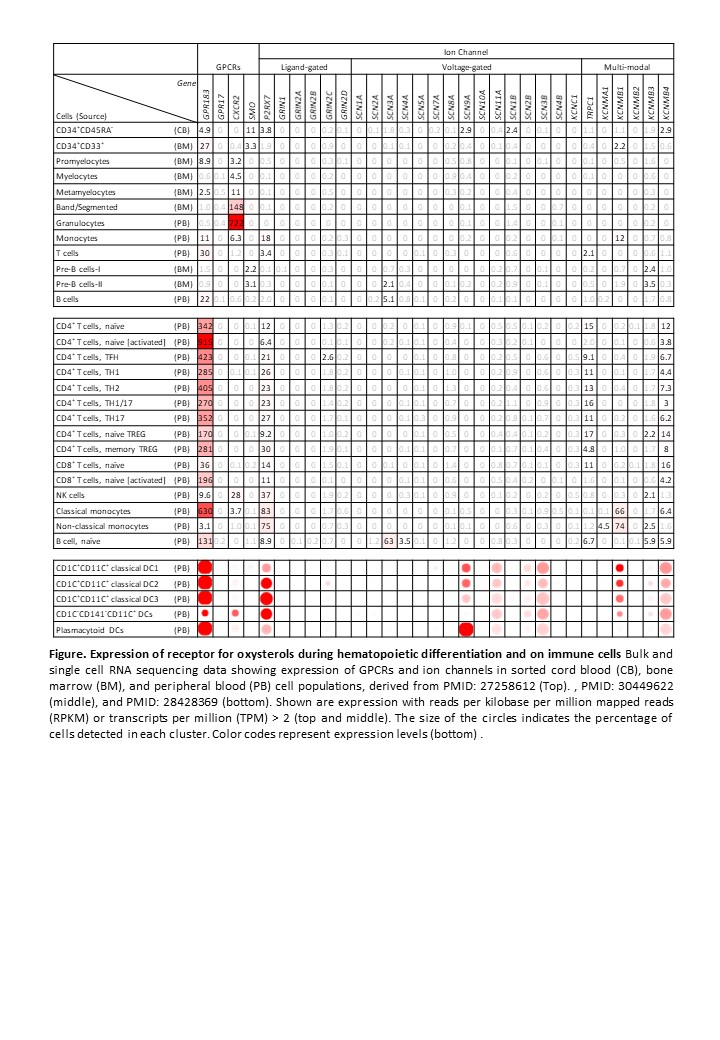

Supplement: Supplementary file 1 [file cells-10-02078-s001.zip › Supplemental Figure 1 V2.jpg]
